# Dynamics of distribution and efficacy of different spot-on permethrin formulations in dogs artificially infested with *Dermacentor reticulatus*

**DOI:** 10.1186/1756-3305-4-45

**Published:** 2011-03-30

**Authors:** Johanna Lüssenhop, Wolfgang Bäumer, Manfred Kietzmann, Thomas Schnieder, Sonja Wolken

**Affiliations:** 1 Institute for Parasitology, University of Veterinary Medicine Hannover, Foundation, Buenteweg 17, 30559, Hannover, Germany; 2 Department of Pharmacology, Toxicology and Pharmacy, University of Veterinary Medicine Hannover, Foundation, Buenteweg 17, 30559, Hannover, Germany

## Abstract

**Background:**

Varying reports concerning the duration and reliability of different permethrin preparations' efficacy can be found in the literature. The aim of this study was to investigate the dynamics of the distribution and efficacy of four different spot-on formulations with permethrin as the active ingredient formulated with different solvents. To examine the influence of these solvents on the speed of distribution and the acaricidal activity of permethrin in the coat, an *in vivo *study under laboratory conditions was performed. Six dogs per test period were treated with the recommended dose and 1, 14 and 28 days after treatment dogs were infested with *Dermacentor reticulatus *ticks: a) on the back, near the application site, and b) on the hind leg, the greatest possible distance from the application site. Efficacies were determined 6 hours after tick infestation to examine the repellent effect and the speed of kill of the products which plays an important role in the context of tick transmitted diseases.

**Results:**

After six hours of exposure, a significant acaricidal efficacy (p < 0.001) could be observed in all treated groups over the whole duration of the study, regardless of which product was used. While the arithmetic mean of attached ticks was < 3 on Day 1, numbers increased over the course of the study to a mean of > 9 on Day 28. However, most of these ticks were dead even 28 days after treatment, as the mean of live attached ticks was still < 2. Significant differences could neither be found between the different permethrin spot-on formulations, nor between the two parts of the body (p > 0.05).

**Conclusions:**

All products were able to kill ticks within six hours following infestation from Day 1 to Day 28 after treatment. Additionally, no significant difference between the tick numbers on the back and the hind leg could be found at any time, which implies a homogenous distribution of permethrin over the body. The efficacy of all four products was comparable during the whole study period, showing that the different solvents do not significantly affect the dynamics of distribution.

## Background

There are currently almost 900 known tick species, of which about 10% are parasites that can affect domestic and companion animals making them of focus in acaricidal control [1,2]. The importance of these arthropods arises especially from their role as vectors and parasites. Ticks are known to transmit viruses, bacteria, fungi, protozoa and nematodes, with every genus and species possessing its own specific germ flora. One of the most important ticks in companion animals is *Dermacentor reticulatus*, a three host tick from the family Ixodidae which is widely distributed across Europe. *D. reticulatus *has been found from southern France to central Germany [3-5], and from the United Kingdom to as far east as Central Asia [6]. *D. reticulatus *achieves its main significance from being a vector of *Babesia canis*, a protozoan piroplasmid that causes babesiosis in dogs [7,8]. Furthermore, *D. reticulatus *transmits *Francisella tularensis*, *Coxiella burnetti*, *Theileria equi *and several *Rickettsia *species [7,9,10].

To minimize the risk of tick transmitted diseases, a compound should ideally prevent tick attachment or should kill attached ticks rapidly. Although the minimal transmission time is hard to assess, it is known for some pathogens that several hours of attachment are required before transmission occurs [11,12]. Since its discovery in the 1970 s [13], permethrin has been used for the prevention and control of many arthropod species. It is a type I pyrethroid that is used in companion and food-producing animals as well as for the impregnation of clothes and nets in order to repel and kill insects and ticks likewise. There are currently four spot-on preparations for dogs registered in Germany which all contain permethrin as the active substance and recommend the same amount of drug per kilogram body weight. However, they all have different pharmaceutical formulations and use varying solvents. A previous study has shown significant differences in the efficacy of two formulations with the same concentration of permethrin dissolved in different solvents [14]. This indicates that solvents may play a role in the distribution of the active ingredient and may therefore influence the efficacy of the drug. In a further study, an increased number of ticks could be observed on the legs of treated dogs in comparison to the rest of the body [15]. The authors explain this with a possible uneven distribution of the active ingredient over the body surface after topical application. However, it has to be kept in mind that this study was a field study which was conducted with dogs that showed varying coat lengths, which can play a role in the distribution of permethrin.

The present study was designed to comparatively evaluate the efficacy of all four permethrin products under laboratory conditions. The efficacy on the hind legs was compared to the efficacy on the back close to the application site at 24 hours, 14 days and 28 days after application. This allowed the evaluation of possible variations in efficacy, which could be related to differences in the distribution of the active substance over the body surface, and of the length of the acaricidal efficacy.

## Methods

### Study design

Four products containing the active substance permethrin were tested in four test periods over a duration of 18 months. Each test period lasted 28 days, starting with the treatment on study day 0 and including experiments on study day 1, 14 and 28 after treatment. To ensure uniform conditions, the study was carried out in a cross-over-design, as every product was tested on the same six dogs at different time points and the control group was formed by the same six dogs in every test period. Furthermore, every product was tested once in the spring and once in autumn in order to take seasonable fluctuations into account, which can even play a role under laboratory conditions. A detailed table of the study design is depicted in Additional File 1. At least 12 weeks were present between two treatments in order to avoid accumulation of the active substance. The four spot-on preparations used in this study were Exspot^® ^(Intervet Deutschland GmbH), Fletic^® ^(IDT Biologika GmbH), Preventic^® ^(Virbac Tierarzneimittel GmbH) and Advantix^® ^(Bayer Vital GmbH). All formulations contain the pyrethroid permethrin in an equal amount per kg of body weight when applied as indicated in the package insert. The solvents contained in the different formulations were 1-methoxypropan-2-ol in Exspot^®^, N-methylpyrrolidon in Fletic^®^, isopropylmyristat in Preventic^® ^and 1-methyl-2-pyrrolidone in Advantix^®^. Advantix^® ^additionally contains the active substance imidacloprid and represents a combination preparation.

The animal experiments have been approved by the federal state authority (AZ. 33.9-42502-04-08/1560).

### Dogs and Ticks

The study was conducted with a group of twelve beagles, which consisted of two females and ten males aged between ten months and 3.5 years. Six of the dogs represented the control group and were treated at no time with permethrin or any other ectoparasiticide. The other six dogs were further divided into two treatment groups of three dogs each. During each test period the dogs were kept in individual pens to rule out any possibility of cross-contamination. Humidity in the rooms ranged between 30 and 70% and temperature ranged between 15 and 25°C.

The study was carried out using unfed adult ticks of the species *D. reticulatus*. The ticks originated from a laboratory reared strain from ClinVet International (Bloemfontein, South Africa) and were classified as pathogen free. One day before each study day, ticks were assessed for activity and presorted into batches of 20, comprising ten males and ten females.

### Treatment

The cross-over design resulted in two products each being tested at the same time in three dogs. Furthermore, each formulation was tested once in the spring and once in autumn. The control group consisted of the same 6 dogs in each test period. Exspot^® ^and Fletic^® ^were tested in spring of year one and autumn of year two. The values of the control animals in these test periods are represented by control I. Preventic^® ^and Advantix^® ^were tested in autumn of year one and spring of year two. The values of the control animals of these test periods are represented by control II. Therefore the simultaneously tested treatments Exspot^®^/Fletic^® ^and Preventic^®^/Advantix^® ^each had their own pool of control animals for comparison.

On study day 0, the dogs of the treatment groups were treated with the products of the current test period. The spot-on was applied with a pipette directly onto the skin after parting the hair between the shoulder blades. The application site was inspected for local reactions 1 hour and 24 hours after treatment.

To take account of the varying weights of the dogs and in order to make all products comparable, the dosage was calculated according to each dog's body weight. The manufacturers of Exspot^®^, Fletic^® ^and Preventic^® ^recommend a dosage of 1 ml (containing 744 mg permethrin) for dogs weighing up to 15 kg, which implies that animals weighing 15 kg receive the minimal effective dosage. This means a minimal dosage of 0.07 ml spot-on, or 49.6 mg permethrin per kg of body weight. Advantix^® ^contains 500 mg permethrin per ml and 2.5 ml of the product are recommended for dogs weighing 10 kg to 25 kg, hence a dosage of 0.1 ml spot-on or 50 mg permethrin per kg of body weight is registered. Therefore, all products were applied with the same amount of active substance per kg body weight.

### Chambers

Infestation with ticks was done using plastic chambers firmly attached to the infestation site on the thoracic wall (close to the permethrin application site) and to the hind leg.

**Figure 1:**
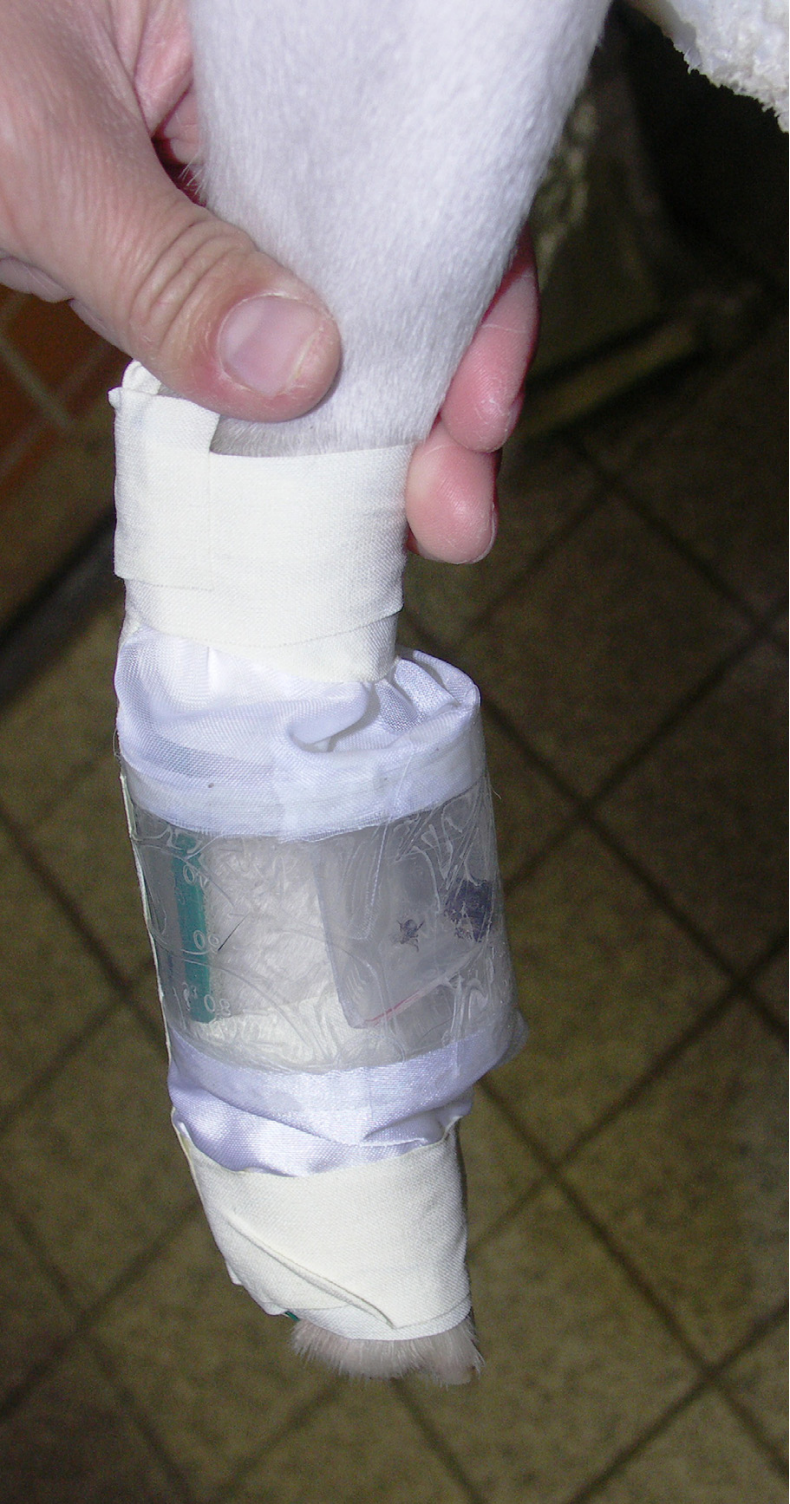
**Leg chamber**. The picture shows the right hind leg of a dog with the leg chamber. The leg splint can be seen in green and the polypropylene tube in the middle with white strips of cloth. They are both fixed to the leg with adhesive tape. The ticks are visible in the retreat area.

Each leg-chamber (Figure 1) consisted of a polypropylene screw cap beaker with a capacity of 120 ml. The thread and bottom were sawed off so as to obtain a tube with a length of 5 cm and a diameter of 5.15 cm. Two 4 cm wide strips of cloth were glued to each opening of the tube around its whole circumference as a means of fixing the chamber around the dog's leg while still maintaining flexibility. This also ensured sufficient air supply for the ticks. To offer the ticks an area for retreat, a piece of bent polypropylene was put into the chamber with its convex side inwards. To keep the chamber in position it was fixed with adhesive tape to a leg splint that was attached to the dog's hind leg. The ticks could be placed in the chamber before the upper side of the tube was sealed.

**Figure 2:**
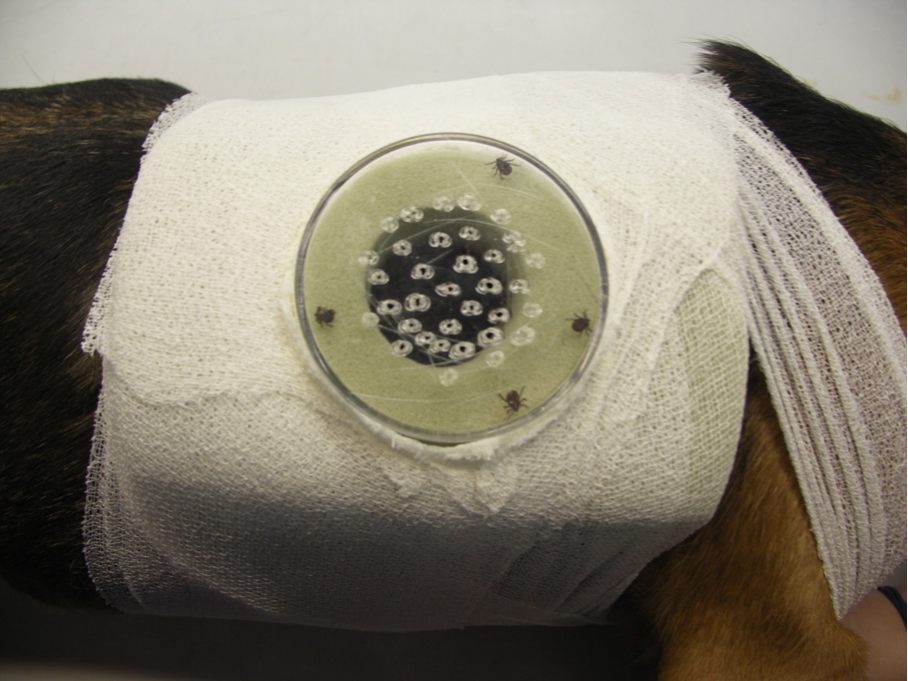
**Back chamber**. The picture shows a back chamber of a dog. The green foam is fixed with white adhesive tape around the thorax. The holes for air supply in the Petri dish are visible as well as the ticks in the retreat area.

Each back-chamber (Figure 2) consisted of a rectangular piece of foam with a size of 15 × 10 × 3 cm. A tunnel with a diameter of 3.5 cm was cut into the middle. The bottom part of a 6.5 cm Petri dish in which 30 small holes had been drilled for air supply was used to close the external opening of this tunnel. The whole appliance was attached to the side of the dog's thoracic wall about 10 cm away from the application site by means of a cohesive elastic bandage. The ticks could then be placed in the chamber by removing the Petri dish.

Both chambers contained a separate compartment where the ticks were placed for infestation. This ensured the application of the ticks without placing them directly onto the dog's fur and without immediate contact to the permethrin which would allow them to escape the repellent activity and stay away from the skin. For their blood meal the ticks had to move actively onto the dog's skin.

### Experiments

Experiments were performed on day 1, 14 and 28 after treatment. Every dog was anaesthetised for approximately 30 minutes with a combination of medetomidinhydrochlorid (0.04 mg/kg of body weight) and ketamin (2 mg/kg of body weight) which was applied intramuscularly. Chambers were then attached to the dog's right side and right hind leg respectively as described above.

The previously assorted ticks were assessed for activity directly before infestation. Each chamber was equipped with 20 ticks and was then sealed to prevent the ticks from leaving their designated skin area. A space collar was fastened around each dog's neck for the time of tick exposure to prevent the animals from damaging the chambers.

### Assessment of ticks

After an exposure period of six hours, the chambers were removed and the ticks were counted and their condition assessed. Each tick was categorized as being either "free" or "attached". Attached ticks were then carefully removed, assessed according to their vitality and categorized as "live" (normal directed movement), "dead" (no movement at all) or "moribund" (ticks showing some kind of movement but not directed). All ticks were assessed again after 24 hours to assure that recovery could not be observed for ticks that had shown no directed movement. As all "moribund" ticks were dead after 24 hours, these were eventually added to the category "dead". The engorgement status was not determined as the time period of six hours was too short for the intake of visible amounts of fluid.

### Statistical methods

For the statistical analysis, data from the spring and autumn were pooled for each product. To evaluate the acaricidal efficacy of the spot-on products after an exposure time of six hours, the total number of dead ticks (free plus attached) in each chamber was analysed. The data for the dead ticks were evaluated with one-way ANOVA and Dunnett's multiple comparison test to compare each treatment group at each time point with its particular control group in order to detect significant differences (p ≤ 0.05). It was further differentiated between extremely significant (***, p < 0.001), very significant (**, p = 0.001 to 0.01) and significant (*, p = 0.01 to 0.05) results. To compare the different products with each other, values of the treated groups were corrected for the values of the respective control. The number of dead ticks in the control group was assumed to be the number of ticks that died from natural causes, so for each dog in each treatment group the arithmetic mean of dead ticks in its specific control group was subtracted from its own number of dead ticks. A Kruskal-Wallis-Test was then performed with these corrected values in order to compare the different products to each other.

To describe the repelling properties of the spot-on products, the total number of attached ticks, regardless of their vitality, was evaluated. This represents a very conservative approach, assuming that every tick attachment harbours the risk of pathogen transmission and not taking the time of feeding into account. To link these data to the acaricidal efficacy, the vitality status of the attached ticks was assessed. Due to the partially low attachment rates in the controls (e.g. control II had a lower attachment rate than the treatment groups on the back on study day 14 and 28) no statistical analysis comparing numbers of total attached ticks in the treatment groups with those in the control groups could be done, however, treatment groups were statistically compared to each other. In order to do this, a correction of the values was performed to take the different control groups into account. It was assumed that the number of attached ticks in the control group represented the attachment rate under the conditions of the experiments, so the arithmetic mean of each control group was used to calculate a relative value. The number of attached ticks of each dog in the treatment group was divided by its particular control group and hereby a relative value which could be used in a Kruskal-Wallis test for the comparison of the different products was generated.

A Mann-Whitney test was performed for the comparison of the data for the leg and back for each product individually.

Statistical analyses were done using GraphPad Prism Version 5.01 (GraphPad Software, La Jolla, USA).

## Results

**Figure 3:**
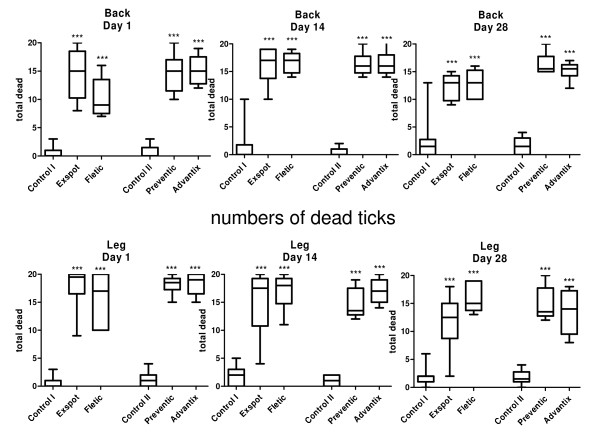
**Dead ticks on the back and on the leg over 28 days**. The figure shows the numbers of dead ticks on the back and leg for each product and for their particular control groups in a box plot diagram. The median is shown as well as the 25% and 75% percentile, and the minimum and maximum number of dead ticks. The total number of ticks applied was 20, the number of dogs was n = 6 for each treatment group and n = 12 for each control group, *** = p < 0.001.

**Figure 4:**
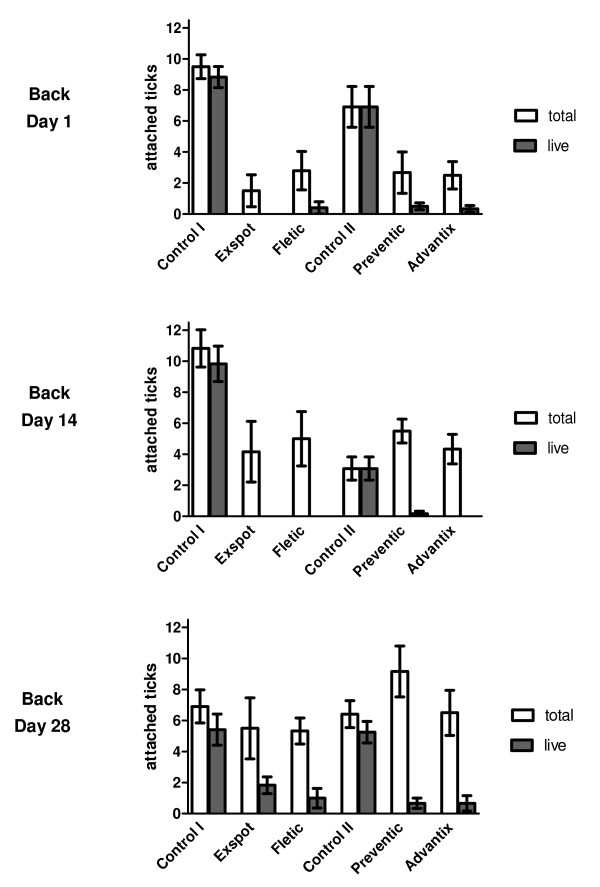
**Attached ticks on the back**. Figure showing the attached ticks on the back over the course of the study. The arithmetic mean of the total of attached ticks (white colour) and of the live attached ticks in particular (dark colour) can be seen for each product and for their particular control groups. The standard error of mean is included.

**Figure 5:**
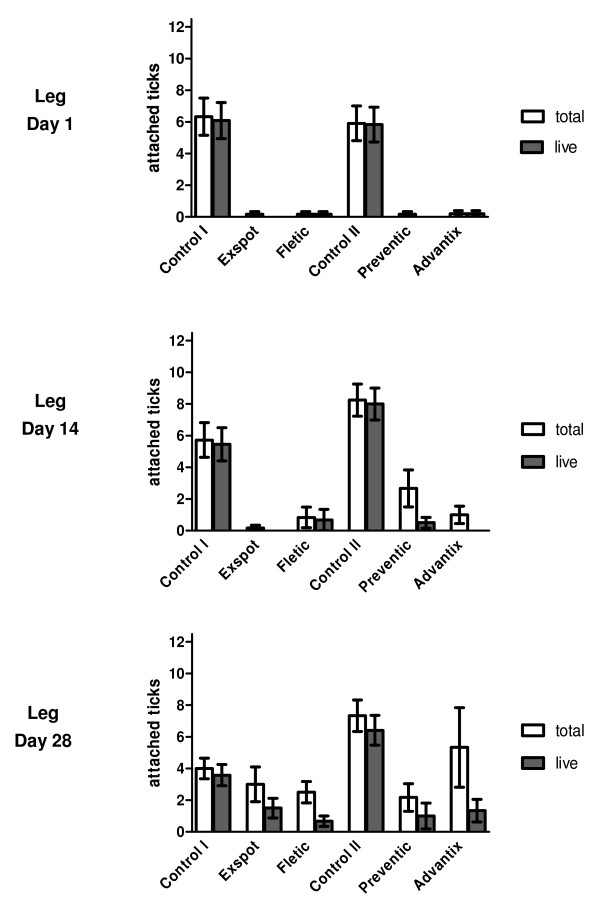
**Attached ticks on the leg**. Figure showing the attached ticks on the leg over the course of the study. The arithmetic mean of the total of attached ticks (white colour) and of the live attached ticks in particular (dark colour) can be seen for each product and for their particular control groups. The standard error of mean is included.

Results of the acaricidal efficacy evaluation are presented as box plots in Figure 3. For graphic presentation of the dead and live attached ticks, the medians are shown as stacked columns in Figure 4 and 5. Individual group values of attached ticks are provided in Additional File 2.

### Acaricidal effect

During the whole study the control groups showed a median of 0 - 1.5 and an arithmetic mean (AM) of 0.4 - 2.2 dead ticks on the back (Figure 3). The permethrin products' median range of dead ticks was 9.0 - 15.0 (AM: 10.2 - 15.2) on Day 1, 16.0 - 17.0 (AM: 16.2 -16.7) on Day 14 and 13.0 - 15.5 (AM: 12.3 - 16.3) on Day 28. This represents an extremely significant (p < 0.001) difference for dead ticks for each product compared to its control group over the whole 28 days. The products were not significantly different to each other (p > 0.05). The statistical analysis for dead ticks on the leg revealed an equally significant difference compared to controls over the whole study period (Figure 3). While the median of the control groups showed a range of 0 - 2.0 (AM: 0.7 - 2.1) dead ticks during the whole study, the median range for the treatment groups was 17.0 - 19.5 (AM: 15.7 - 18.4) on Day 1, 13.5 - 18.0 (AM: 14.7 - 17.0) on Day 14 and 12.5 - 15.0 (AM: 11.7 - 15.8) on Day 28. No significant difference could be seen between the products.

The statistical analysis for the numbers of dead ticks on the back and leg in comparison for each product individually did not show a significant difference (p > 0.05). Neither did the values of the control groups for the two parts of the body.

### Repellent effect

When assessing the attached ticks on the back over the whole duration of the study, the control groups showed a median range of 3.0 - 11.5 and an AM range of 3.1 - 10.8. In the treatment groups, the median range was 0 - 2.0 (AM: 1.5 - 2.8) attached ticks on Day 1, 3.5 - 5.0 (AM: 4.3 - 5.5) on Day 14 and 4.5 - 9.5 (AM: 5.3 - 9.2) on Day 28. However, most of these ticks were dead, which is illustrated in Figure 4. The median range of attached ticks that were alive was only 0 - 0.5 (AM: 0 - 0.5) on Day 1, 0 (AM: 0 - 0.2) on Day 14 and 0 - 2.0 (AM: 0.7 - 1.8) on Day 28. It is evident that the number of attached ticks increased over the duration of the study from an arithmetic mean of < 3 to > 9. While only single ticks were alive 24 hours and 14 days after treatment, a mean of up to 2 live attached ticks could be found on the last study day.

For the live attached ticks on the back, no significant difference between the products could be seen (p > 0.05). For the numbers of total attached ticks, a significant difference was present between Exspot^®^/Fletic^® ^and Preventic^®^/Advantix^® ^on Day 14 on the back (p = 0.002).

On the leg, the control groups showed a median range of 4.0 - 8.5 and an arithmetic mean range of 4.0 - 8.3 attached ticks over the whole duration of the study (Figure 5). Compared to this, the treatment groups showed a median of 0 attached ticks on Day 1 (AM: 0.2), 0 - 2.0 (AM: 0.2 - 2.7) on Day 14 and 2.0 - 4.0 (AM: 2.2 - 5.3) on Day 28. Like on the back, the numbers of attached ticks that were still alive on the leg were a lot lower, with a median of 0 (AM: 0 - 0.2) on Day 1, 0 (AM: 0 - 0.7) on Day 14 and 0 - 1.5 (AM: 0.7 - 1.5) on Day 28. Just like on the back, an increase of attached ticks towards the end of the study is apparent while the increase of attached ticks that were still alive was not as distinct. There was no significant difference between the numbers of attached ticks on the leg for the different products (p > 0.05).

The comparison of the numbers of live attached ticks on the leg and back for each product individually did not show a significant difference (p > 0.05), nor did the comparison of the numbers for the control groups.

## Discussion

The four products examined in this study have, to our knowledge, never been tested under equal conditions before. Many factors during a study can influence the results in various directions. Examples are the seasonal and even daily fluctuations in tick activity, or the constitution of the laboratory animals. Deviating study designs can also induce varying results [16,17]. Consequently, it is difficult, or even impossible, to compare the results of studies which have been performed in different laboratories, and at different times. If the characteristics of several different products are compared, it is crucial to test them under similar conditions. This was achieved in the current study as all products were tested under laboratory conditions on the same dogs in a cross-over design over a period of 18 months.

Furthermore, the study design enables distinction between the effect of the products in different areas of the body, i.e. close to and far away from the application site. Previous studies have reported a higher concentration of ticks on the legs of treated dogs, which was explained by an uneven distribution of the active ingredient [15]. To examine this observation more closely, chambers were built for the back and for the hind leg in order to evaluate independently the effect of permethrin in the two areas.

The tick species used in the study was *D. reticulatus*. Due to its role as a vector of *B. canis*, it belongs to the most important tick species in Europe. However, very few efficacy studies have been carried out with *D. reticulatus*. Furthermore, most acaricides are not explicitly tested and registered for their efficacy against this tick species meaning that only very limited data is available. *D. reticulatus *was therefore chosen for this study to generate data concerning the efficacy of the available permethrin products against this important tick. According to our own experience with acaricides, *Dermacentor *seems to be less sensitive towards acaricidal products compared to other genera such as *Ixodes*. It may therefore be assumed that a product which effectively repels or kills *Dermacentor *will also perform favourably with other tick species.

In order to evaluate the acaricidal efficacies of the products, the number of dead ticks was assessed after an exposition time of six hours. This time period was chosen according to pilot experiments which were carried out for up to 12 hours. The results show a significant efficacy of all products already 24 hours after treatment on the back as well as the leg. This indicates that all formulations distributed the permethrin over the whole body surface within 24 hours following topical application. The effect of the active ingredient remained apparent over the whole study period in all treatment groups, as the numbers of dead ticks were still significantly higher after 28 days. Furthermore, the speed of kill was very high, as the ticks were only exposed to the product for six hours. According to the "Guideline for the Testing and Evaluation of the Efficacy of Antiparasitic Substances for the Treatment and Prevention of Tick and Flea Infestation in Dogs and Cats" by the EMEA [18], ticks are recommended to be assessed 24-48 hours after tick infestation, hence in this study an even higher speed of kill could be determined than that necessary for the registration of a product. The Guideline further advises including the engorgement status of the ticks in the interpretation of data, but this was not possible due to the short exposure period of six hours.

Products with repelling properties, such as the investigated permethrin products, strive to protect the treated animal against attachment of ticks. This study demonstrates that this can not be completely achieved by any of the products over the duration of 28 days. However, the specific study design has to be kept in mind, as the ticks were trapped in their chambers. Under natural conditions repellency is an immediate effect that usually makes the tick leave the host after coming into contact with a repelling substance. Although the chambers contained a separate compartment for the ticks to hide and possibly escape the acaricide, it may be possible that after being forced to stay on the animal, the temptation of a blood meal was stronger than the repellent effect of the drug. On the other hand, it has been reported that permethrin is not only absorbed after direct contact, but that concentrations in the air can lead to the excretion of metabolites in exposed people [19]. This potential acaricidal effect on ticks that have not come into contact with the hair or the skin has to be kept in mind as the possibility of a certain permethrin concentration in the air can not be eliminated in the chambers. Also, the anaesthesia which was performed on the dogs has to be considered when assessing the behaviour of ticks. The changes in metabolism during anaesthesia and the lacking movement of the dogs differ from natural conditions. This may affect ticks concerning their movement and their desire to attach to the host. It can generally be assumed that it is easier for a tick to attach when the host is immobile.

Although the presence of attached ticks is generally to be avoided, most of the attached ticks in this study were dead six hours after infestation, hence they were killed within less than six hours after contact with the host. Some studies have been conducted concerning the times of feeding which are necessary for the transmission of particular diseases [11,12]. They indicate that the risk of transmitting diseases is very low when a tick has only been feeding for a few hours, but for some pathogens exact minimal transmission times still have to be investigated, for example for *Anaplasma phagocytophilum *and different *Rickettsia *species. This would be very valuable information concerning the estimation of the efficacy of repelling and acaricidal products. When comparing the numbers of attached ticks of the different products, no significant difference between the products could be found, except for the back on Day 14. Here the groups Exspot^®^/Fletic^® ^and Preventic^®^/Advantix^® ^were significantly different from each other. This can be explained by the very low tick numbers of the control group of Preventic^® ^and Advantix^®^, which was used to calculate a relative value to be able to compare the groups with each other. The absolute numbers of the different products were very similar on that day.

When comparing the data for the leg with that for the back, no major differences were found. When the control groups were taken as a reference, the two areas of the body generally showed slightly different numbers of attached ticks, which implies that the back region is principally more appealing to the ticks. However, reduction of attached ticks in comparison to the control was the same for both parts of the body. This leads to the assumption that the concentration of the active ingredient permethrin was comparable on the back and leg even after four weeks implying that the distribution of permethrin over the skin is homogenous, whether the area is close to the application site or as far away as possible on the hind leg.

## Conclusions

The study shows that the different solvents in the four examined products do not lead to a different distribution of permethrin over the body surface as all the products show a homogenous effect at the different body parts over 28 days. In all products efficacy decreases over the study period of 28 days and there is no significant difference between the four products' efficacy and speed of kill.

## Competing interests

The authors declare that they have no competing interests.

## Authors' contributions

SW, WB, MK and TS participated in the conception and design of the study. SW coordinated the study. JL carried out the experimental part of the study and the statistical analysis and drafted the manuscript. WB participated in analyzing the data.

All authors read and approved the final manuscript.

## Supplementary Material

Additional file 1 **Study design**. Table showing the four study periods including seasonal information and the allocation of the dogs to the different products and the two control groups. Click here for file

Additional file 2 **Data for attached ticks**. Table showing data of the total of attached ticks and the live attached ticks. The range of found ticks is shown for each product for all study days as well as the arithmetic mean and median. Click here for file
