# Supplementary material for: Dynamics of distribution and efficacy of different spot-on permethrin formulations in dogs artificially infested with Dermacentor reticulatus
Source: Parasit Vectors. 2011 Mar 30;4:45. doi: 10.1186/1756-3305-4-45 (PMC3073924; doi:10.1186/1756-3305-4-45)
Supplement: Additional file 1 — Study design. Table showing the four study periods including seasonal information and the allocation of the dogs to the different products and the two control groups. [file 1756-3305-4-45-S1.PDF]

Study Design	treatment (dog 1 – 3)	treatment (dog 4 – 6)	control (dog 7-12)
**spring 2009**	Exspot®	Fletic®	control I
**autumn 2009**	Preventic®	Advantix®	control II
**spring 2010**	Advantix®	Preventic®	control II
**autumn 2010**	Fletic®	Exspot®	control I
