# Supplementary material for: Dynamics of distribution and efficacy of different spot-on permethrin formulations in dogs artificially infested with Dermacentor reticulatus
Source: Parasit Vectors. 2011 Mar 30;4:45. doi: 10.1186/1756-3305-4-45 (PMC3073924; doi:10.1186/1756-3305-4-45)
Supplement: Additional file 2 — Data for attached ticks. Table showing data of the total of attached ticks and the live attached ticks. The range of found ticks is shown for each product for all study days as well as the arithmetic mean and median. [file 1756-3305-4-45-S2.PDF]

		Day 1		Day 14		Day 28	
		**back**	**leg**	**back**	**leg**	**back**	**leg**
**Control I**	range attached total	6 - 15	1 - 14	2 - 16	0 - 12	1 - 12	0 - 9
	range attached live	6 - 14	1 - 14	2 - 15	0 - 12	0 - 11	0 - 9
	arithm. mean attached total	9.5	6.33	10.83	5.73	6.92	4.0
	arithm. mean attached live	8.83	6.08	9.83	5.46	5.42	3.58
	median attached total	9.0	5.5	11.5	5.0	6.0	4.0
	median attached live	8.5	5.0	10.5	5.0	5.5	3.5
**Exspot®**	range attached total	0 - 6	0 - 1	0 - 11	0 - 1	0 - 13	0 - 7
	range attached live	0	0	0	0	0 - 4	0 - 4
	arithm. mean attached total	1.5	0.17	4.17	0.17	5.5	3.0
	arithm. mean attached live	0	0	0	0	1.83	1.5
	median attached total	0	0	3.5	0	4.5	3.5
	median attached live	0	0	0	0	2.0	1.5
**Fletic®**	range attached total	0 - 7	0 - 1	0 - 12	0 - 4	3 - 8	0 - 4
	range attached live	0 - 2	0 - 1	0	0 - 4	0 - 4	0 - 2
	arithm. mean attached total	2.8	0.17	5.0	0.83	5.33	2.5
	arithm. mean attached live	0.4	0.17	0	0.67	1.0	0.67
	median attached total	2.0	0	3.5	0	5.5	3.0
	median attached live	0	0	0	0	0.5	0.5
**Control II**	range attached total	1 - 16	0 - 14	0 - 8	3 - 13	3 - 14	1 - 13
	range attached live	1 - 16	0 - 14	0 - 8	3 - 13	3 - 12	1 - 12
	arithm. mean attached total	6.92	5.92	3.08	8.25	6.42	7.33
	arithm. mean attached live	6.92	5.83	3.08	8.0	5.25	6.42
	median attached total	6.0	6.0	3.0	8.5	6.0	8.0
	median attached live	6.0	5.5	3.0	8.5	4.5	6.5
**Preventic®**	range attached total	0 - 9	0 - 1	4 - 9	0 - 8	3 - 15	0 - 5
	range attached live	0 - 1	0	0 - 1	0 - 2	0 - 2	0 - 5
	arithm. mean attached total	2.67	0.17	5.5	2.67	9.17	2.17
	arithm. mean attached live	0.5	0	0.17	0.5	0.67	1.0
	median attached total	1.5	0	5.0	2.0	9.5	2.0
	median attached live	0.5	0	0	0	0.5	0
**Advantix®**	range attached total	0 - 5	0 - 1	1 - 8	0 - 3	2 - 12	0 - 17
	range attached live	0 - 1	0 - 1	0	0	0 - 3	0 - 4
	arithm. mean attached total	2.5	0.2	4.33	1.0	6.5	5.33
	arithm. mean attached live	0.33	0.2	0	0	0.67	1.33
	median attached total	2.0	0	4.5	1.0	6.0	4.0
	median attached live	0	0	0	0	0	0.5
